# Factors Associated With the Uptake of Cervical Cancer Screening Among Family Medicine Physicians, Compared With Women of the Community in Riyadh, Saudi Arabia

**DOI:** 10.7759/cureus.53283

**Published:** 2024-01-31

**Authors:** Hamdan D Alshehri, Ola Alqudah, Firas B Almadani, Ahmed M Aldalbahi, Omar a Jarrah, Ahmad Albashaireh

**Affiliations:** 1 Family Medicine, King Fahad Medical City, Riyadh, SAU; 2 Nursing, Faculty of Health Sciences, Higher College of Technology, Fujairah, ARE

**Keywords:** saudi females, kingdom of saudi arabia (ksa), cervical cancer screening, cervical screening, family medicine, pap test

## Abstract

Background: Cervical cancer is the ninth diagnosed cancer among Saudi women. The majority of cervical cancer cases occur in women who did not undergo screening. However, the screening rates in several countries, including Saudi Arabia, remain suboptimal. It is important to identify the factors associated with the uptake of screening and predictors of screening in order to increase the uptake rate.

Aim: To determine the factors associated with the uptake of cervical cancer screening among family medicine physicians (FMPs), compared with women of the community.

Methods: This was a cross-sectional study conducted in the central region (Riyadh), Kingdom of Saudi Arabia from February 2021 for 12 months on female physicians and women of the community. An electronic questionnaire was used to investigate the demographics of women and variables related to the uptake of screening.

Results: A total of 126 FMP and 127 women from the community were included. The factors affecting screening among FMP included age (P=0.013), health insurance (P=0.002), availability of Pap smear (P˂0.001), and physician encouragement (P˂0.001). The factors affecting the screening of community women included the availability of Pap smears (P˂0.001) and physician encouragement (P˂0.001). Multivariate analysis revealed that physician encouragement of Pap smear was a significant predictor of screening among FMP (OR=8.26, P˂0.001) and community women (OR=6.67, P˂0.001). The perceived benefit was the only predictor for screening among FMP (OR=0.75, P=0.004).

Conclusion: The uptake of cervical cancer screening was higher in the community women. The factors linked to the uptake differed among the two groups, but the support of doctors played a significant role in the likelihood of uptake, regardless of the group of women. It is recommended to enhance the guidance of medical personnel in recommending screening during clinic visits for the specific target group. Additionally, there should be increased education on the significance of screening and efforts to educate the community about cervical cancer and screening.

## Introduction

Cervical cancer ranks fourth among the most common cancers in females [1]. Almost 570000 women were diagnosed with cervical cancer in 2018, and almost 311000 women died globally [1]. In 2020, in Saudi Arabia, the crude incidence of cervical cancer per 100000 women was 2.4, and the mortality due to cervical cancer rated 0.5 [1]. Cervical cancer in Saudi Arabia ranks as the ninth most commonly diagnosed cancer among women aged 15-44 years [2]. The majority of new cervical cancer cases (83%) and related mortality (85%) occur in middle and low-income groups, affecting vulnerable, poor, and disenfranchised women at the prime of life [3].

The majority of cervical cancer cases occur in women who did not undergo screening [4]. In Saudi Arabia, the factors contributing to the increased incidence of cervical cancer include the lack of a structured national screening program, the increased prevalence of human papillomavirus, and social and behavioral factors [5].

In order to reduce the incidence and mortality of cervical cancer, the United States (US) Preventive Services Task Force recommended screening for women aged 21-65 years every three years [4]. A meta-analysis reported that the mortality risk due to cervical cancer was 35%, and this was a lower rate found among women invited to screening with cytology tests compared to women who were not offered screening [6].

However, the screening rates in several countries, including Saudi Arabia, remain suboptimal [7,8]. The average proportion of cervical cancer screening in developing nations is 19%, whereas, in developed nations, it is 63% [9]. One study reported that only 16.8% of women from Jeddah performed the Pap screening [7]. A more recent study in 2019 reported the screening rates among women in different countries, including Saudi Arabia was found that screening rates were 7.6% in Saudi Arabia, 17.7% in Kuwait, 10.6% in Oman, and 28% in the United Arab Emirates (UAE) [8].

Several international studies identified the predictors and barriers to the uptake of cervical cancer screening. A study from Peru revealed that accepting a Pap smear was affected by socioeconomic status, lack of appropriate counseling, low income, and embarrassment or fear during the screening procedure [10]. In Uganda, it was found that higher screening rates were found among women who were wealthy, professional, had four or more children, and had a high level of knowledge about cervical cancer and screening, as well as women who obtained their information from healthcare providers [11].

In a study from Iran, there were personal barriers identified for a second Pap smear, and they included inattention to time and inhibiting beliefs. Also, negative experience with the first Pap test was another barrier [12]. The factors associated with the uptake of screening and predictors of screening have not been compared between women from the community and female physicians as family medicine physicians are the first line to see patients and we would like to see if this screening is reflected in family medicine physicians themselves and find out the different factors between the two groups. Therefore, we conducted the current study, and as far as we know, this is the first study report on this subject.

## Materials and methods

Study design, setting, and participants

This study deployed a descriptive cross-sectional design. This study was conducted in the central region (Riyadh) of the Kingdom of Saudi Arabia. Females from family medicine and community were enrolled in this study to determine the factors associated with the uptake of cervical cancer screening.

The target population included married women aged 21 to 65 who resided in Riyadh. Furthermore, family medicine physicians (FMP) had to be consultants, specialists, or residents. Women who had had hysterectomies with cervix removal, were unmarried, aged less than 21 or above 65, were not living in Riyadh, and any participants who were not eager to participate were excluded from the study. A nonprobability convenience sample of 253 (126 FMP, 127 community women) women were successfully recruited. 

Data collection procedure

Data was collected from February 2021 for 12 months from primary healthcare centers and communities in the central region (Riyadh) of the Kingdom of Saudi Arabia. Data were collected using an electronic survey by combining all the study instruments in one questionnaire. The approved survey by the ethical committees was communicated and distributed to all healthcare facilities to invite potential participants of FMP to participate in the study. Also, researchers approached community women and asked them to participate in the study. Participants were asked to give their consent before participation. The survey remained distributed over the period of data collection, and all responses were received by the study supervisor and saved in a secured area until the time of analysis.

Data collection tool

A survey comprised of three major sections was used to collect the data from the participants. The first section includes general sociodemographic information such as age, marital status, level of education, employment, nationality, and health insurance coverage (see Appendix A).

The second section asked participants about the current services, such as the availability and follow-up for Pap smear and cervical cancer. They were also asked if they had ever undergone cervical cancer screening (see Appendix B).

The third section used a reliable and valid questionnaire that has four scales: perceived benefits (5 items), barriers (10 items), susceptibility (4 items), and seriousness (6 items). Each item is measured via a four-point Likert scale ranging from 4 (strongly agree) to 1 (strongly disagree). In all scales, a higher score represents a higher level of perceived benefits, barriers, susceptibility, and seriousness of screening, and the lowest score represents minimal benefits, barriers, susceptibility, and seriousness of screening. A study by Al-Amro et al. (2020) reported a good internal consistency for this questionnaire with Cronbach's alpha of 0.78 [13]. In this study, Cronbach's alpha was 0.83 (see Appendix C).

Ethical considerations

This study was approved by the Institutional Review Board (IRB) of King Fahad Medical City (KFMC), Riyadh, Kingdom of Saudi Arabia. Participation in this study was entirely voluntary, and participants were free to withdraw at any point without jeopardizing their rights or any reason. Participation was anonymous, and participants were asked to read the participation information sheet and sign the consent form before completing the self-reported questionnaires. All data were used only for this research study. The researcher secured all collected data through a secure protective device to avoid any breach of confidentiality.

Data analysis

The statistical software Statistical Package for Social Sciences (SPSS), version 22.0 (IBM Corp. Armonk, NY) was used to analyze the data. All data were screened for abnormalities and missing; then analyses were conducted on the clean data. All nominal and ordinal data were reported in frequency percentages. Continuous quantitative data were reported in mean and standard deviation. Chi-squared test or Fisher’s exact test was used to check for differences or associations for the nominal variables. An independent t-test was used to compare the differences in means of the total score for perceived benefits, barriers, susceptibility, and perceived seriousness of cervical cancer between the two groups of family medicine and community groups. Binary logistic regression was used to identify the predictors for the uptake of cervical cancer screening in family medicine and community groups.

## Results

This study enrolled 253 participants from two groups of women, 126 family medicine physicians, and 127 community women without any medical background. The two groups were similar regarding age groups, marital status, and health insurance coverage. The uptake of cervical cancer screening was higher in the community women. The majority of family medicine physicians and community women were below 40 years old (73% and 66.2%, respectively), married (89.7% and 80.3%, respectively), and without health insurance (64.4% and 70.9%, respectively) (Table 1). The two groups differed in terms of level of education, employment, and nationalities. Compared with community women, the family medicine group had a higher employment rate (96% vs. 15.3%,respectively) and a higher level of education (100% vs. 15.3%, respectively); but with a lesser number of Saudi nationalities (84.9% vs. 96.9%, respectively) (Table 1).

**Table 1:** Sample characteristics ¶ Chi-squared or Fisher’s exact test * Statistically significant at (α ≤ 0.05), ** Statistically significant at (α ≤ 0.01), *** Statistically significant at (α < 0.001)

Characteristics	Family Medicine N = 126 n (%)	Community N = 127 n (%)	p-value^¶^
Age			0.167
21-30 years	51 (40.5)	35 (27.6)	
31-40 years	41 (32.5)	49 (38.6)	
41-50 years	25 (19.8)	29 (22.8)	
51-65 years	9 (7.1)	14 (11.0)	
Marital status			0.105
Married	113 (89.7)	102 (80.3)	
Separated	12 (9.5)	22 (17.3)	
Widowed	1 (0.8)	3 (2.4)	
Level of education			< 0.001***
Below secondary level	0 (0.0)	6 (4.7)	
Secondary level	0 (0.0)	16 (15.3)	
Higher education	126 (100.0)	105 (15.3)	
Employment			< 0.001***
Employed	121 (96.0)	89 (70.1)	
Not employed	3 (2.4)	34 (26.8)	
Retired	2 (1.6)	4 (3.1)	
Nationality			0.001**
Saudi	107 (84.9)	123 (96.9)	
Non-Saudi	19 (15.1)	4 (3.1)	
Health insurance coverage			0.264
Yes	45 (35.7)	37 (29.1)	
No	81 (64.3)	90 (70.9)	

Table 2 shows participants' responses toward their Pap smear experiences. Only 42.1% of the FMP and 22.8% of the community reported Pap smear availability in the health sector (p < 0.001). Compared with the community group, the family medicine group reported a higher rate of the influence of healthcare follow-up on undergoing the exploratory Pap smear (75.4% vs. 42.5%, respectively, p < 0.001). Both groups were similar in reporting the suitability of the Pap smear's waiting period, the contribution of medical recommendation for acceptance of Pap smear, and the presence of a male physician as obstacles for performing Pap smear (Table 2). Two groups differed in reporting if physicians encouraged them to have a Pap smear and if they ever had a cervical Pap smear; such differences were not significant but close to the significant level. The community group had a higher rate of ever having a cervical pap smear than the family medicine group (30.7% vs. 20.6%, respectively, p = 0.067) (Table 2).

**Table 2:** Participants’ responses toward their Pap smear experiences ¶ Chi-squared or Fisher’s exact test * Statistically significant at (α ≤ 0.05), ** Statistically significant at (α ≤ 0.01), *** Statistically significant at (α < 0.001)

Variables	Family Medicine N = 126 n (%)	Community N = 127 n (%)	p-value^¶^
Is Pap smear available in the health sector?			< 0.001***
Yes	53 (42.1)	29 (22.8)	
No	46 (36.5)	26 (20.5)	
I didn’t ask	27 (21.4)	72 (56.7)	
Does the existence of healthcare follow-up encourage you to undergo an exploratory Pap smear?			< 0.001***
Yes	95 (75.4)	54 (42.5)	
No	31 (24.6)	73 (57.5)	
Is the waiting period for a cervical smear suitable?			0.774
Yes	24 (19.0)	24 (18.9)	
No	12 (9.5)	9 (7.1)	
Not undergone this service before	90 (71.4)	94 (74.0)	
Did the physician encourage you to have a Pap smear?			0.056
Yes	49 (38.9)	35 (27.6)	
No	77 (61.1)	92 (72.4)	
Is the presence of a physician (a man) an obstacle to performing a Pap smear?			0.083
Yes	45 (35.7)	59 (46.5)	
No	81 (64.3)	68 (53.5)	
Are the medical recommendations contributing to encouraging your acceptance of a Pap smear?			0.409
Yes	108 (85.7)	104 (81.9)	
No	18 (14.3)	23 (18.1)	
Have you ever had a cervical Pap smear?			
Yes	26 (20.6)	39 (30.7)	0.067
No	100 (79.4)	88 (69.3)	

Table 3 compares perceived benefits, barriers, susceptibility, and perceived seriousness for family medicine and community groups. There were significant differences in the mean of the total score for perceived benefits of cervical cancer screening (p = 0.002) and the perceived susceptibility to cervical cancer (p = 0.001) between the family medicine group and the community. The two groups were similar regarding the perceived barriers to cervical cancer screening and the perceived seriousness of severity of cervical cancer (Table 3).

**Table 3:** Perceived benefits, barriers, susceptibility, and perceived seriousness among participants ¶ Independent t-test * Statistically significant at (α ≤ 0.05), ** Statistically significant at (α ≤ 0.01), *** Statistically significant at (α < 0.001)

Variables	Mean ± SD	t-value	p-value^¶^
Total perceived benefit to cervical cancer screening			
Family medicine (n =126)	16.32 ± 2.86	-3.064	0.002**
Community (n = 127)	17.33 ± 2.37		
Total perceived barrier to cervical cancer screening			
Family medicine (n =126)	19.83 ± 4.37	-1.058	0.291
Community (n = 127)	20.43 ± 4.76		
Total perceived susceptibility to cervical cancer			
Family medicine (n =126)	9.67 ± 2.41	-3.352	0.001**
Community (n = 127)	10.65 ± 2.24		
Total perceived seriousness of severity of cervical cancer			
Family medicine (n =126)	17.40 ± 3.62	1.464	0.144
Community (n = 127)	16.80 ± 2.77		

Table 4 compares perceived benefits, barriers, susceptibility, and perceived seriousness between those who had a Pap smear and those who never had a Pap smear in the family medicine and community groups. Further, those who had a Pap smear and who never had a Pap smear were compared across the two groups. In the family medicine group, there are only significant differences in the mean of total perceived benefits between those who had a Pap smear and those who never had a Pap smear (18.04 ± 2.13 vs. 15.87 ± 2.87, p < 0.001). The community group had no significant differences in all four perceived parameters. Across the groups, there were significant differences in the mean of total perceived benefits to cervical cancer screening (15.87 ± 2.87 vs. 17.17 ± 2.42, p = 0.001) and the perceived susceptibility to cervical cancer (9.48 ± 2.36 vs. 10.68 ± 2.21, p < 0.001) for those who never had a Pap smear (Table 4).

**Table 4:** Comparison of perceived benefits, barriers, susceptibility, and perceived seriousness among participants within the group (ever and never had Pap smear) and between the two groups (family medicine and community women) ¶ Independent t-test ^ t and p-values were not given in the table * Statistically significant at (α ≤ 0.05), ** Statistically significant at (α < 0.01), *** Statistically significant at (α < 0.001)

	Family Medicine (n =126) (26 had Pap smear, 100 never had Pap smear)			Community (n = 127) (39 had Pap smear, 88 never had Pap smear)			Family Medicine vs. Community (had vs. had, never vs. never)
Variables	Mean ± SD	t-value	p-value^¶^	Mean ± SD	t-value	p-value^¶^	p-value^¶^^
Total perceived benefit to cervical cancer screening							
Had Pap smear	18.04 ± 2.13	3.604	< 0.001**	17.69 ± 2.26	1.144	0.255	0.538
Never has Pap smear	15.87 ± 2.87			17.17 ± 2.42			0.001**
Total perceived barrier to cervical cancer screening							
Had Pap smear	19.12 ± 4.43	-0.930	0.354	20.82 ± 5.45	0.610	0.543	0.189
Never has Pap smear	20.01 ± 4.35			20.26 ± 4.44			0.696
Total perceived susceptibility to cervical cancer							
Had Pap smear	10.42 ± 2.50	1.796	0.075	10.59 ± 2.33	-0.213	0.832	0.784
Never has Pap smear	9.48 ± 2.36			10.68 ± 2.21			< 0.001***
Total perceived seriousness of severity of cervical cancer							
Had Pap smear	18.23 ± 4.18	1.181	0.246	16.79 ± 2.87	-0.022	0.982	0.105
Never has Pap smear	17.18 ± 3.45			16.81 ± 2.74			0.410

Table 5 compares responses of two groups of family medicine and community responses about possible factors that could influence their uptake of the Pap smear. Two groups differed in reporting if family members, friends, husbands, religion, or culture encouraged them to have a Pap smear; such differences were statistically significant (all p < 0.001). The highest reinforcing reported reinforcing factor in family medicine and community groups was religion, followed by culture (Table 5).

**Table 5:** Reinforcing factors for Pap smear ¶ Chi-squared or Fisher’s exact test * Statistically significant at (α ≤ 0.05), ** Statistically significant at (α ≤ 0.01), *** Statistically significant at (α < 0.001)

Characteristics	Family Medicine N = 126 n (%)	Community N = 127 n (%)	p-value^¶^
Family members encourage me to have a Pap smear			< 0.001***
Never	97 (77.0)	51 (40.2)	
Sometimes	23 (18.3)	54 (42.5)	
Most of the time	4 (3.2)	9 (7.1)	
Always	2 (1.6)	13 (10.2)	
My friends encourage me to decide to have a Pap smear			< 0.001***
Never	87 (69.0)	55 (43.3)	
Sometimes	24 (19.0)	42 (33.1)	
Most of the time	12 (9.5)	14 (11.0)	
Always	3 (2.4)	16 (12.6)	
My husband agrees and encourages me to have a Pap smear			< 0.001***
Never	76 (60.3)	44 (34.6)	
Sometimes	24 (19.0)	50 (39.4)	
Most of the time	19 (15.1)	9 (7.1)	
Always	7 (5.6)	24 (18.9)	
The Islamic religion encourages and promotes health prevention behavior, including conducting a Pap smear			0.001**
Never	50 (39.7)	24 (18.9)	
Sometimes	17 (13.5)	37 (29.1)	
Most of the time	22 (17.5)	9 (7.1)	
Always	37 (29.4)	57 (44.9)	
Our culture, as well as customs and traditions, supports the decision to have a Pap smear			< 0.001***
Never	73 (57.9)	38 (29.9)	
Sometimes	35 (27.8)	37 (29.1)	
Most of the time	6 (4.8)	20 (15.7)	
Always	12 (9.5)	32 (25.2)	

Table 6 illustrates factors associated with the uptake of Pap smear among FMP and community groups. In the FMP group, age (p = 0.013), health insurance coverage (p = 0.002), availability of Pap smear in the health sector (p < 0.001), medical recommendations for acceptance of Pap smear (p = 0.023), and physician encouragement to undergo the Pap smear screening (p < 0.001) were significantly associated with the uptake of Pap smear (Table 6). In the community group, the availability of Pap smear in the health sector (p < 0.001) and physician encouragement to undergo the Pap smear screening (p < 0.001) were significantly associated with the uptake of Pap smear (Table 6). 

**Table 6:** Factors associated with the uptake of Pap smear among participants ¶ Chi-squared or Fisher’s exact test * Statistically significant at (α ≤ 0.05), ** Statistically significant at (α ≤ 0.01), *** Statistically significant at (α < 0.001)

	Family Medicine (N = 126) (26 had Pap smear, 100 never had Pap smear)			Community (N = 127) (39 had Pap smear, 88 never had Pap smear)		
Variables	Had Pap smear n (%)	Never had Pap smear n (%)	p-value^¶^	Had Pap smear n (%)	Never had Pap smear n (%)	p-value^¶^
Age			0.013*			0.051
≤ 40 years	14 (53.8)	78 (78.0)		21 (53.8)	63 (71.6)	
> 40 years	12 (46.2)	22 (22.0)		18 (46.2)	25 (28.4)	
Marital status			0.467			0.092
Married	22 (84.6)	91 (91.0)		35 (89.7)	67 (76.1)	
Separated/Widowed	4 (15.4)	9 (9.0)		4 (10.3)	21 (23.9)	
Employment			0.583			0.779
Employed	24 (92.3)	97 (97.0)		28 (71.8)	61 (69.3)	
Not employed/Retired	2 (7.3)	3 (3.0)		11 (28.2)	27 (30.7)	
Has health insurance			0.002**			0.264
Yes	16 (61.5)	29 (29.0)		14 (35.9)	23 (26.1)	
No	10 (38.5)	71 (71.0)		25 (64.1)	65 (73.9)	
Is Pap smear available in the health sector?			< 0.001***			< 0.001***
Yes	20 (76.9)	33 (33.0)		18 (64.2)	11 (12.5)	
No	5 (19.2)	41 (41.0)		7 (17.9)	19 (21.6)	
I didn’t ask	1 (3.8)	26 (26.0)		14 (35.9)	58 (65.9)	
Did the physician encourage you to have a Pap smear?			< 0.001***			< 0.001***
Yes	20 (76.9)	29 (29.0)		22 (56.4)	13 (14.8)	
No	6 (23.1)	71 (71.0)		17 (43.6)	75 (85.2)	
Is the presence of a physician (a man) an obstacle to performing a Pap smear?			0.088			0.468
Yes	13 (50.0)	32 (32.0)		20 (51.3)	39 (44.3)	
No	13 (50.0)	68 (68.0)		19 (48.7)	49 (55.7)	
Are the medical recommendations contributing to encouraging your acceptance of a Pap smear?			0.023*			0.143
Yes	26 (100.0)	82 (82.0)		35 (89.7)	69 (78.4)	
No	0 (0.0)	18 (18.0)		4 (10.3)	19 (21.6)	

The factors for the uptake of cervical cancer screening in all participants (FMP and community) and in the group of family medicine and community were displayed in Table 7. In all participants, the group, age, perceived benefits, and physician encouragement to undergo cervical screening were predictors for cervical cancer screening. In the family medicine group, perceived benefits and physician encouragement to undergo cervical screening are the predictors for cervical cancer screening. In the community group, only the physician's encouragement to undergo cervical screening is the predictor for cervical cancer screening. In both groups, physician encouragement increases the likelihood of uptake for cervical cancer screening (OR = 8.26, p ˂ 0.001; OR = 6.67, p ˂ 0.001, respectively). In the family medicine group, a one-unit increment in the total score of perceived benefit increases the likelihood of the uptake of cervical cancer screening (OR = 0.75, p = 0.004) (Table 7). 

**Table 7:** Binary logistic regression for the factor for uptake of cervical cancer screening among participants * Statistically significant at (α ≤ 0.05), ** Statistically significant at (α ≤ 0.01), *** Statistically significant at (α < 0.001)

	All Participants N = 253			Family Medicine N = 126			Community N = 127		
Variables	p-value	OR	95% CI (Lower-Upper)	p-value	OR	95% CI (Lower-Upper)	p-value	OR	95% CI (Lower-Upper)
Group (family medicine vs. community)	< 0.033*	0.45	(0.24-0.94)						
Age (≤ 40 vs. > 40)	< 0.031*	0.48	(0.25-0.94)	0.095	0.41	(0.14-1.17)	0.233	0.59	(2.25-1.41)
Physicians encourage Pap smear (yes vs. no)	< 0.001***	7.02	(3.58-13.77)	< 0.001***	8.26	(2.80-24.36)	< 0.001***	6.67	(2.77-16.06)
Perceived benefit	< 0.006**	0.829	(0.73-0.95)	0.004**	0.75	(0.61-0.91)	0.425	0.93	(0.77-1.12)

## Discussion

There was no previous Saudi study comparing the factors affecting the uptake of cervical cancer screening between female physicians and women from the community. In our study, the rate of uptake of Pap smear among the FMP group was surprisingly lower compared to that of the community women; the uptake of the FMP group was 20.6%, whereas that among women of the community was 30.7%. A previous Saudi study reported that 66.6% of women from Jeddah did not have a Pap test, whereas only 33.4% underwent the smear [14]. 

A community-based study from Ethiopia found that the uptake of cervical cancer screening was only 3% [15]. The findings in our study were better, where 33.4% of women from the community underwent a Pap smear. However, our findings were less compared to another community-based cross-sectional study from Ethiopia there, where 38.7% screened for cervical cancer [3]. A study in Ghana reported a very low rate of having a Pap smear, where only 0.8% of 392 participants reported having a Pap smear [16]. These findings were much worse compared to ours. 

There was no previous Saudi study that reported the uptake of smears among female physicians; however, we surprisingly found that the rate among the physician population was lower compared to the community women. Additional findings revealed that women from the community significantly had a higher mean of perceived benefits to screening and perceived susceptibility compared to the FMP group. 

Furthermore, having a Pap smear had significant impacts, where those who had a Pap smear reported a significantly higher mean of perceived benefit to cervical cancer screening in the FMP group only, whereas such impact was not significant in the women community group. Additionally, the mean of perceived benefits of those who had the smear among the FMP group was significantly higher compared to women from the community.

A previous Saudi study from Jeddah was conducted on 385 women aged 21-65 years living in Jeddah. The univariate analysis revealed that the factors significantly associated with the screening status of having a Pap smear or not included education level, increased age, monthly income, source of information, having a family doctor, and perceived risk of getting cervical cancer [14].

The factors affecting the uptake of the smear varied between the two major groups (FMP and women of the community groups) and between those who underwent the smear and those who did not. For the FMP group, age, health insurance, availability of Pap smears in the health sector, and encouragement of the physician were factors affecting the uptake of Pap smears and medical recommendations. However, the uptake of Pap smear among community women was significantly affected by the availability of Pap smear in the health sector, and the encouragement of the physician were factors affecting the uptake of Pap smear only. Additionally, such later two factors significantly varied between the FMP group and the community group.

Due to these variations in factors, logistic regression was done to determine the factors of uptake of cervical cancer screening. It was found that physician encouragement of the smear increased the probability of uptake among the FMP group and community group by more than twofold. The perceived benefit was a predictor for uptake among the FMP group only. All such findings indicate that the determination of factors affecting the uptake of screening should be conducted individually on each population of females as the factors vary greatly between different women, even in some countries, based on the community they belong to.

A community study from Ethiopia revealed that the factors for the uptake of cervical cancer included marital status (AOR = 10.74), residence (AOR = 4.45), educational status (AOR = 1.95), distance from health facility (AOR = 4.41), health workers encouragement (AOR = 3.23), awareness on cervical cancer (AOR = 0.37), awareness about cervical cancer screening (AOR = 4.52) and number of health facility visit per year (AOR = 3.63) [3]. In contrast to the previous findings, our study revealed that the uptake of women in the community was not associated with age, marital status, or employment. However, in logistic regression, the uptake of women was increased due to physician encouragement, and this was in agreement with the previous study.

A community-based study revealed that knowing the availability of cervical cancer screening services was significantly associated with the uptake of screening (AOR=2.8) [17]. In our study, the availability of Pap smears in the health sector was significantly associated with the uptake of Pap smears in both groups. Nonetheless, this factor was not a predictor for the uptake of the smear based on logistic regression.

In Jamaica, the predictors of screening uptake included age, being married, perception of consequences of not having the smear, and discussing cancer with the health provider [18]. A study from Jordan demonstrated that the encouragement of healthcare providers (OR=5.24), years of marriage (OR=1.09), and the use of the private healthcare sector (OR=2.2) were significant predictors of cervical cancer screening [13]. The previous two studies revealed the importance of healthcare providers in the uptake of Pap smears. Our study also reinforces such findings, as the encouragement by physicians to undergo the screening was a significant predictor for the uptake of screening. 

One study investigated the factors affecting the uptake of cervical cancer screening among medical lecturers, and only 79 respondents were included. Of them, more than one-half (55.7%) had ever undergone a Pap smear. The uptake of Pap smear was significantly associated with age (P=0.001) and level of education (P=0.003). The perception of not being at risk (22.9%) was a reason for not performing the Pap smear [19]. The rate of uptake in the previous study was better compared to ours regarding the uptake of Pap smear among the FMP group. Also, in contrast to the previous study, age was not associated with the uptake of the smear.

A systematic review from Nigeria aimed to assess the factors impacting cervical cancer screening among female healthcare workers included 15 studies. It was found that the uptake of cervical cancer screening was poor. The authors categorized the factors into barriers and facilitators; the barriers included low-risk perception, lack of test awareness, and the cost of screening, whereas the facilitating factors included increasing age, being married, physician recommendation, and awareness of screening methods [20]. 

Strengths and limitations 

The present research has a number of limitations. First, the use of cross-sectional precludes the cause-and-effect relationships between the variables. Moreover, convenience sampling, which was used in our research, can be one of the limitations because of its nonprobability sampling technique, which cannot represent all of the population. As a result, skewed findings and incorrect conclusions can result. Another possible limitation is reporting bias because of using self-reported questionnaires. Furthermore, the sample size was relatively small. Despite such limitations, the sample size was adequate and was unique since it first compared community women with family medicine physicians.

## Conclusions

The uptake of cervical cancer screening varied among different female groups, where more women in the community tended to uptake the screening rather than family medicine physicians. Additionally, the factors associated with uptake varied between the two groups. However, encouragement of physicians could be an important predictor for uptake, regardless of the women's group. Guidance of medical staff should be augmented to recommend screening for targeted groups at clinic visits, more education regarding the importance of screening should be imparted, and campaigns undertaken to educate the community about cervical cancer and screening.

## Appendices

Appendix A

We prepared the following form to study the factors associated with the uptake of cervical cancer screening among family medicine physicians compared to the community in Riyadh, Kingdom of Saudi Arabia.

Socio-Demographic Data

1) Your age in years:

2) Current marital status:

- Married

- Divorced

- Widowed

3) Nationality:

- Saudi

- Non-Saudi

4) Are you a doctor:

- Yes

- No

5) Level of education:

- Less than secondary school

- Secondary school

- Higher education.

6) Current working status:

- Employed

- Not employed

- Retired

Enabling Factors Questionnaire

Please answer the following questions. If you have any questions, please ask the researcher.

1) Do you have health insurance?

- Yes

- No

2) Is there cervical cancer screening services (Pap test) in your health sector?

- Yes

- No

- Never asked

3) Having a regular source for health care encouraged you to uptake a cervical cancer screening tests (Pap smear):

- Yes

- No

4) Clinic time (time waiting to get the service) uptake of cervical cancer screening (Pap smear) is convenient:

- Yes

- No

5) Did health care providers, encourage you for cervical cancer screening uptake (Pap test):

- Yes

- No

6) Presence of male physician prevents you from Pap test uptake:

- Yes

- No

- If answer “yes,” specify:

7) Physician’s recommendation for cervical cancer screening (Pap test uptake) increases the likelihood of doing it:

- Yes

- No

Cervical Cancer Screening (Pap Smear Uptake)

Have you ever had a cervical cancer screening (Pap smear)?

- Yes

- No

1) If “yes,” how many times:

2) If “no,” indicate why you never had a cervical cancer screening (Pap smear):

- Because I never heard of it

- I do not feel it is necessary

- I am scared of the procedure

- I do not know where I can get it

- My religion/culture does not permit it

- I do not feel at risk for cervical cancer

- Absence of female physician in clinic.

- I have no time

-I feel embarrassment

Appendix B

**Table 8:** Predisposing factors questionnaire (attitude toward screening) Indicate the extent to which you agree or disagree with the following statements reflecting your own personal opinion regarding each statement (4=strongly agree; 1=strongly disagree)

NO	Items	Strongly agree	Agree	I don’t know	Disagree	Strongly disagree
1. Perceived benefits of cervical cancer screening						
1	It is important for a woman to have cervical cancer screening to know if she is healthy.	4	3	0	2	1
2	Cervical cancer screening can find changes in the cervix before they become cancerous.	4	3	0	2	1
3	If cervical changes are found early from cervical cancer screening, they are easily curable.	4	3	0	2	1
4	Doing cervical cancer screening can help improve the chances of an infertile woman becoming pregnant.	4	3	0	2	1
5	Cervical cancer screening can decrease the chance of a woman having an abortion.	4	3	0	2	1
2. Perceived barriers to cervical cancer screening						
1	It is too embarrassing to do cervical cancer screening.	4	3	0	2	1
2	Cervical cancer screening is painful.	4	3	0	2	1
3	Doing cervical cancer screening will make one worry.	4	3	0	2	1
4	Only women who have had babies need to do cervical cancer screening.	4	3	0	2	1
5	Not knowing where to go for cervical cancer screening is a reason why people don’t do cervical cancer screening.	4	3	0	2	1
6	My husband will not want me to do cervical cancer screening.	4	3	0	2	1
7	Lack of female screeners in health facilities is a reason for not doing cervical cancer screening.	4	3	0	2	1
8	Attitudes of health workers can discourage one from going for cervical cancer screening.	4	3	0	2	1
9	Lack of information about cervical cancer screening procedures is a barrier to the uptake of cervical cancer screening.	4	3	0	2	1
10	Lack of convenient clinic time is a barrier to routine cervical cancer screening.	4	3	0	2	1
3. Perception about susceptibility to cervical cancer						
1	Older women are more at risk of cervical cancer than younger women.	4	3	0	2	1
2	Every woman of childbearing age is at risk of cervical cancer.	4	3	0	2	1
3	Women with multiple sexual partners are more prone to cervical cancer.	4	3	0	2	1
4	Susceptibility to cervical cancer increases with the number of pregnancies.	4	3	0	2	1
5	Cervical cancer only happens to women who are above the age of 50 years.	4	3	0	2	1
4. Perception about the seriousness of severity of cervical cancer						
1	There is an effective treatment for cervical cancer.	4	3	0	2	1
2	Having cervical cancer will make women’s life difficult.	4	3	0	2	1
3	Cervical cancer is not as serious as other types of cancer.	4	3	0	2	1
4	Cervical cancer is easily cured.	4	3	0	2	1
5	Having cervical cancer can result in infertility.	4	3	0	2	1
6	Death resulting from cervical cancer is rare.	4	3	0	2	1

Appendix C

**Table 9:** Reinforcing factors questionnaire Indicate whether you consider the following statement as (never=1; to some extent=2; most of the time=3; always=4)

No	Items	Never	To some extent	Most of the time	Always
1	My family encourages me to do a cervical cancer screening (pap smear).	1	2	3	4
2	My friends facilitated my decision to do cervical cancer screening (pap smear).	1	2	3	4
3	My husband approves a cervical cancer screening (pap smear test).	1	2	3	4
4	Islamic teachings support preventive measures such as screening for cervical cancer (pap smear).	1	2	3	4
5	Arab cultural norms support the decision for cervical cancer screening uptake (pap smear).	1	2	3	4
